# Concussion management in US college football: progress and pitfalls

**DOI:** 10.2217/cnc.15.6

**Published:** 2015-08-06

**Authors:** Christine M Baugh, Emily Kroshus

**Affiliations:** 1 Interfaculty Initiative in Health Policy, Harvard University, 14 Story Street, Fourth Floor, Cambridge, MA 02138, USA; 2 Division of Sports Medicine, Boston Children’s Hospital, 300 Longwood Avenue, Boston MA 02115, USA; 3 Harvard School of Public Health, Department of Social & Behavioral Sciences, 677 Huntington Avenue, Kresge 7th Floor, Boston, MA 02116, USA

**Keywords:** American football, college, concussion, health education, health policy, public health

## Abstract

Reducing the frequency and severity of concussions from sport is an important issue in public health currently addressed by a multifaceted approach. Given the large number of participants and the comparatively high risk of injury, American football is an important sport to consider when examining concussion management practices. Focusing on American football at the collegiate level, this manuscript describes current research regarding concussion epidemiology, policy, implementation of clinical diagnosis, management and return-to-play standards and athlete concussion education. Although American collegiate sports leagues have put forth concussion-related policies in recent years, the implementation of these policies and related effects on athlete concussion education, clinical management of concussion and ultimately athlete health outcomes are not well understood. Additional research is needed.

In recent years, concussion from sport has received tremendous attention from lay and scientific audiences alike, with a frequent focus on concussions sustained as a result of participation in American football (henceforth referred to as ‘football’). Approximately three million youth, one million high school [1], over 70,000 collegiate athletes [2] participate in football annually in the USA. The attention on concussions in football may be warranted, as of all sports involving contact or collision, football presents among the highest risk of concussive injury for its participants [3]. In this manuscript we focus on concussions and concussion management in collegiate football, focusing on policy, clinical practice, concussion education and areas of improvement. Although we focus on football, a significant portion of the material applies to other contact or collision sports, particularly at the collegiate level.

Characterizing the frequency of concussion is usually done in reference to the number of participant exposures to a game or practice – what is called an ‘athletic exposure’ or AE. Estimates of concussion injuries ranged between 0.57 and 1.16 per 1000 AEs across two seasons of competition in a study by Kilcoyne and colleagues [3]. Gessel *et al*. [4] estimated that 0.61 concussions per 1000 AEs occurred in their sample of college football players, and Dompier *et al*. [5] found 0.83 concussions per 1000 AEs in a more recent examination of injuries in college football athletes. Using NCAA Injury Surveillance System data, Dick and colleagues found that rates of concussive injury during practices and games also vary between the spring and fall seasons [6]. Recent estimates suggest that the risk of concussion is much higher in football games than in practice across all levels of competition; however, the total number of concussions sustained in practice is greater than the number sustained in games, due to the greater frequency of practices than games during the season [5]. Notably, the rates of concussion during games were significantly greater at the college level (3.74 concussions per 1000 AEs) than at the youth or high school levels (2.38 and 2.01 concussions per 1000 AEs, respectively) [5]. At the college level, concussions sustained during practices in which athletes are fully padded (wearing all protective equipment) and scrimmages account for a disproportionately large percentage of all concussions sustained during practices [7]. This goes against previous research which suggested that helmets-only practices were associated with greater impacts than games or scrimmages [8]. However, given that the most severe concussions were sustained during practices where athletes wore shells; more research related to equipment type and concussion risk is warranted [7].

Positional activities vary greatly in football, and consequently the nature of head impacts and risk of concussion is not uniform across all team members; this has been demonstrated across multiple research modalities [9–21]. The American Medical Society for Sports Medicine has identified playing position as an important consideration in any examination of concussion among football athletes [22]. Consistent with their role in frequent but short distance contact, offensive and defensive linemen have been found to receive the greatest number of impacts but tend to incur high magnitude impacts less frequently than skill positions [9–12]. Conversely, skill positions tend to receive less frequent impacts but tend to receive more high magnitude impacts than the linemen, likely resultant from their roles in the open field [9–12]. A substantial literature has utilized accelerometers within football helmets to characterize the magnitude and direction of impacts sustained by athletes in different playing positions [10–13]. Similarly, studies using clinical examination or self-report of concussion have found variation in concussive injuries across playing positions [13–21]. A recent survey-based study characterized concussion and concussion-related outcomes in a cohort of NCAA Division I football players and found that offensive linemen reported more frequent suspected but undiagnosed concussions, ‘dings’, and a variety of postimpact symptoms [21]. Much of this literature utilizes self-report of concussion, which recent research indicates may be unreliable [23]. As such, additional research in this area may be needed.

Concussions present both acute and possible chronic manifestations. Acutely, concussions can cause a variety of physical, cognitive, mood and behavioral symptoms [24]. Most frequently, concussion symptoms resolve in less than 2 weeks [24]; however, there is the possibility of longer lasting symptoms, particularly for athletes who have suffered at least one concussion prior to their current injury [25]. Additionally, there is a period of metabolic vulnerability during recovery from a concussion, during which sustaining an additional head impact can result in more severe neurological consequences [26]. Furthermore, after sustaining one concussion athletes are at greater risk of sustaining a subsequent concussive injury [27]. Chrisman and Richardson found a threefold increase in diagnosed depression for 12–17-year olds who had suffered at least one prior concussion compared with those who had never sustained a concussion [28]. In recent years, the possible later-life effects of repetitive concussion have become better understood. Repeated concussions have been found to be associated with later life depression [29–32] and executive dysfunction [33] in former football players. Additionally, repetitive head impacts, including concussion, have been associated with a neurodegenerative disease called chronic traumatic encephalopathy (CTE) [34–36]. The pathophysiologic mechanisms linking head impacts to these later-life manifestations is unclear and further research into this area is warranted.

## Policy

In collegiate sport, one mechanism that has been used to attempt to reduce the frequency of concussions and as well as to ensure that athletes who sustain a concussion receive appropriate medical care is league-level rules governing concussion management. Given that state-based concussion statutes do not generally apply to collegiate athletes [37], these league-based policies are important avenues through which athlete health and well-being can be protected. Recently, concussion laws and sports-league policies have been framed as public health policies [38]. That is, concussion policies are examples of policies aimed to identify, prevent or mediate health risks in a given population [38]. Through this lens, the empirical health law research framework [39], an approach for utilizing empirical data to examine laws or policies, can be used to evaluate the efficacy of these policies. Consistent with this framework, this section presents the current understanding of the sports-league concussion policies and existing evidence regarding their implementation and efficacy.

In 2010, the National Collegiate Athletic Association (NCAA) enacted its Concussion Policy and Legislation [40]. This policy, which applies to all member-schools, requires each school to have a concussion management plan and that the plan has four main components:

- Annual athlete concussion education and athlete acknowledgement of their receipt of this information and of their role in reporting concussion symptoms;
- Removal from play for athletes exhibiting concussion signs or symptoms and evaluation of such athletes by medical personnel;
- Preclusion of athletes diagnosed with a concussion from resuming practice or competition for at least the remainder of the calendar day;
- Medical clearance of athletes diagnosed with a concussion by a physician or a physician’s designee prior to returning to athletic practice or competition.

To date, there have been only a handful of investigations of school-level compliance with the NCAA policy. In 2013, Kroshus *et al.* examined the concussion education provided to collegiate ice hockey players [41]. Discussed in more detail later in the education section of this manuscript, this study found that there was wide variation in the nature of the concussion education provided to athletes. Kilcoyne and colleagues examined the rates of concussion diagnosis at three NCAA Division I football programs before and after the NCAA’s rules were implemented [5]. They found a significant increase in rates of diagnosis following the rule change, indicating that the policy may be achieving one of its intended goals; however, given other influences that may have occurred simultaneously (e.g., an overall increase in concussion awareness) causality could not be attributed to the policy change alone [5].

The largest study on the topic to date was a 2014 study by Baugh and Kroshus *et al.* examining compliance with NCAA concussion policy by surveying coaches, clinicians and compliance officers at NCAA member schools [42]. With representation from 85% of NCAA schools, this study found that at the vast majority schools (82%) a concussion management plans was in place. Likewise nearly all respondents indicated they believed that their concussion management plan protected their school’s athletes ‘well’ or ‘very well.’ In line with previous research finding variable implementation of rules and best practice guidelines in sports medicine [43], it was found that despite the broad presence of the concussion management policies at NCAA member schools some of the implementation of the policies’ components lagged behind. For example, just 70% of institutions represented had an annual athlete concussion education process in place. This study also provided insight on areas in which stakeholders felt that improvement was needed related to concussion management. About three-quarters of respondents felt that some improvement was needed at their school. Education of coaches, education of athletes and more staffing in the sports medicine department were the most frequently selected areas in need of improvement. It is important to note that current concussion-related rules at the NCAA level are secondary and tertiary prevention mechanisms – that is, they relate to promptly identifying and appropriately treating concussions that occur, rather than preventing the initial injury.

The NCAA has taken additional steps to address the issue of concussions in college athletes. For example, there have been several football rule changes aimed at reducing concussions such as: moving the starting position for the kickoff from the 30- to the 35-yard line and placing touchbacks on the 25-yard line rather than the 20-yard line both aimed at reducing high magnitude impacts on kickoff and kickoff return [44]. The extent to which these and other rule changes have actually reduced concussions is unknown. Additionally, in 2014 it put forth guidelines including best practices for concussion management plans, clinical management, return to play and return to school [45]. These guidelines also included recommendations limiting contact practices in football [46]. The extent to which these nonbinding guidelines have been implemented at member schools is unclear. However, given the recent literature indicating that the majority of concussions in football are sustained during practice [5], encouraging a reduction in contact sustained during practices is an important step forward.

In setting rules and guidelines the NCAA provides a minimum acceptable standard; divisions of competition and conferences have the opportunity for targeted initiative-taking in the area of concussion and elsewhere. Some conferences have put in place measures specific to their membership aimed at reducing concussions and improving concussion diagnosis and management. For example, the Ivy League and the Pac-12 conferences have put forth restrictions on the amount of contact allowed in practice for football and other sports [47,48]. The Big Ten conference was the first to take the step of putting in place an enforcement mechanism related to its concussion policies: it will penalize schools that do not comply with its enhanced concussion policy [49]. Following a proposal put forth by the Southeastern Conference, the NCAA Committee on Competitive Safeguards recently created a Concussion Safety Protocol committee that will annually review concussion management protocols of the Power 5 Conference schools (a portion of the NCAA Division I) [50].

Other collegiate sports organizations have put forth concussion guidance in recent years. The National Association of Intercollegiate Athletics (NAIA) endorsed new concussion guidelines rules in the 2014–2015 academic year [51]. They recommended that each institution have a written policy to address concussions; though the contents of the policy are not specified and the compliance with the recommendation is not monitored. The National Junior College Athletic Association (NJCAA) proposed development and implementation of a concussion management plan by all member institutions in 2012 [52]. Interestingly, explicitly mentioned in the rationale for this proposal was that the “concussion management plan could also protect the college legally from litigation stemming from injuries, or conversely, open it up to litigation if no such plan is in place,” [52]. The NJCAA recommends consultation with the NCAA Sports Medicine Handbook as well as the National Federation for High School Sports guidance for concussion management plan policy guidelines [52].

Research related to concussion management and the effects of concussion management policies at NAIA and NJCAA levels are sparse. One study by Chinn and Porter examined concussion management at California Community Colleges where a football program was in place [53]. This mixed methods study found varying levels of implementation of best practices of concussion management at these institutions. Similar to the findings at the NCAA level, staffing issues were frequently cited as hindering appropriate management of concussion. Budget constraints or budget cuts were also described as a major issue for concussion management [53].

## Clinical diagnosis & management

At the collegiate level, the sports medicine team is the cornerstone of concussion recognition, diagnosis and management. Recent expert consensus statements put forth guidelines for acute concussion management [22,24,54–55]. Although great strides have been made in the validation of new clinical tools providing a more accurate assessment of concussion, there is no universally accepted, reliable and valid biomarker diagnosis of concussion [56]. Additionally, oftentimes concussion diagnosis and management are reliant upon athletes reporting their postimpact symptoms honestly [56]. Reliance on athlete disclosure of symptoms combined with the heterogeneous presentation of acute symptoms in multiple domains can result in challenges for clinical diagnosis. Best practices in concussion management are well described in the position statements of the National Athletic Trainers’ Association (NATA) [54], American Academy of Neurology [55], American Medical Society for Sports Medicine [22] and the Zurich International Conference on Concussion in Sport [24]. Rather than providing a review of the existing literature on acute concussion diagnosis and management, this section will describe research relating to the implementation of best practice guidelines for concussion management at the collegiate level as well as environmental and interpersonal factors that may affect concussion management in college sports.

Lynall and colleagues surveyed a cross-sectional sample of NATA members about their concussion management practices [57]. The study sample was comprised of athletic trainers at multiple levels of competition, but approximately a third were from the college level [57]. In this mixed sample it was found that while the use of more objective measures such as balance testing and neuropsychological testing had increased compared with previous estimates, the use of clinical evaluation and symptom scores had decreased [57]. The authors describe this finding as problematic, given that a thorough clinical examination is one of the most important components of concussion diagnosis and management. Athletic trainers at the collegiate level, compared with the high school level, more frequently used computerized neuropsychological testing rather than noncomputerized neuropsychological testing. This may reflect budgetary constraints at the lower levels of competition.

A recent study by Kelly *et al.* examined a group of NCAA Division I athletic trainers regarding their practices for baseline examination, concussion evaluation and return to play decision making [58]. In line with existing guidance, the majority of respondents in this study indicated that they used multiple assessment tools at baseline, for acute evaluation and when making return to participation determinations [58]. Balance, symptom assessment and neuropsychological testing were all frequently used for baseline testing and concussion management, according to this group of respondents [58]. Additionally, this study found high awareness of the NCAA Concussion Policy as well as the NATA position statement and the Consensus in Sport Group Consensus Statement. However, despite awareness of these guidelines, adherence to them was less consistent. For example, despite recommendations, fewer than 10% of respondents to the survey indicated that a licensed neuropsychologist evaluated athletes’ neuropsychological test results [58].

Research related to implementation of best practices in concussion management at non-NCAA collegiate leagues is minimal. Chinn and Porter examined concussion management in California Community Colleges that had a football team [53]. In their examination, nearly three-quarters of respondents indicated that they did not perform baseline testing. Symptom checklists were reported as the most frequently used tool for both sideline examination and return to play determination in this sample and were used by approximately 80% of respondents [53]. More objective assessments were used by approximately a third of respondents, with computerized cognitive testing used by only 10% [53].

The Chinn and Porter study [53] highlights a possible discrepancy between the resources and management practices of concussion management at the NCAA level and within their cohort of California Community Colleges. The extent to which this discrepancy generalizes to community colleges or non-NCAA collegiate sports leagues more broadly is unclear, but is an area worthy of exploration. NAIA had 89 schools that sponsored football teams in the 2014–2015 season [59]. The NJCAA and the California Community College system each have approximately 70 sponsored football teams in their league [60,61]. Understanding the management practices at these levels of competition and working toward implementing best practices is essential.

The competitive environment of collegiate athletics may complicate concussion diagnosis and management. In sports contexts in general, and football in particular, playing through pain or injury is often considered normative and is positively reinforced by coaches and teammates [62,63]. Perhaps not surprisingly then, under-reporting of possible concussions by athletes is one major barrier to diagnosis and treatment. A growing body of evidence estimates that at least half of athletes in contact and collision sports have knowingly or unknowingly not reported symptoms of a possible concussion [21,64–73]. The Chinn and Porter study indicated that over half of athletic trainers surveyed felt a moderate amount of pressure from athletes to return to play and around 40% reported experiencing a high degree of pressure from athletes to return to play [53]. Similarly, Kroshus and colleagues found that over half of athletic trainers at NCAA member schools felt pressured by athletes and by coaches to prematurely return an athlete to play following a concussion [74]. In this study, less-experienced clinicians, female clinicians and clinicians under the supervisory purview of the athletic department (rather than a medical institution) were more likely to report feeling pressured [74]. The competitive sports environment may not only affect athletic trainers in their management of concussion. A recent study indicated that football players who perceived greater support from their coach to report a possible concussion were more likely to intend to do so and less likely to have played through possible concussion symptoms in the 2 weeks preceding the survey [75]. Additionally, research in a sample of collegiate athletes in contact and collision sports other than football found that those who experienced pressure from multiple stakeholders in the athletic environment were significantly less likely to intend to report a concussion to a medical professional; it is likely that these results generalize to football [76]. It is critical that future research examines how to combat the competitive pressures of the collegiate sports environment that hinder concussion safety.

## Concussion education

Concussion education has the potential to contribute to the primary, secondary and tertiary prevention of concussions, with different behavioral targets relevant for different stakeholders at each level of prevention. Primary prevention of concussion is best conceptualized as stopping a concussive impact from occurring. Concussion education relevant to primary prevention may be most appropriately targeted at parents and athletes when they are making decisions about sport participation, either beginning participation in a given sport, or continuing participation in a subsequent season. Education for coaches can also target primary preventive behaviors. Coaches may be able to modify the frequency with which their team engages in contact during practices, or the style of play they reinforce. Secondary prevention of harm from concussion may be best conceptualized as ensuring that athletes who sustain a concussive impact are immediately removed from play so that they do not sustain another impact while symptomatic, a period during which the harm of additional brain trauma is magnified [26]. Given the potential for harm and the opportunity for behavioral risk reduction, this is the level of prevention at which most concussion education materials for athletes have been targeted to date. A main behavioral target for this type of concussion education is athletes choosing to report symptoms of a suspected concussion to a responsible adult. Concussion education for parents, coaches and clinicians on the sidelines of sports games similarly aims engage these stakeholders in removing symptomatic athletes from play. Finally, tertiary prevention may be best conceptualized as care postconcussion to minimize harm. Clinicians who provide care to concussed athletes are important recipients of this level of education, with the behavioral target being the nature of the care they provide athletes. Coaches, parents and athletes may also be relevant targets for tertiary prevention-related concussion education, with a focus on increasing adherence to medical directives during the concussion recovery process. Tertiary prevention could also be relevant outside of the immediate sports context. For student-athletes, academic accommodations are often necessary during the concussion recovery process, however there is variable implementation of such accommodations across schools [77]. Education for all school stakeholders, including administrators, teachers and school nurses, has been recommended as one strategy for helping student-athletes successfully navigate academic demands during the recovery process [78].

Consistent with this expansive potential for concussion education, the most recent Consensus in Sport Group Consensus Statement indicates “athletes, referees, administrators, parents, coaches and healthcare providers must be educated regarding the detection of concussion, its clinical features, assessment techniques and principles of safe return to play.” [24]. This message appears to be resonating, at least in the USA, as evidenced by the near ubiquity of concussion education mandates at the state and sports-league level [37]. Nearly all states require that athletes in high school and younger be provided with information about concussions [37]. At the collegiate level, the NCAA mandates that all institutions provide student-athletes with informational materials about concussion on an annual basis [44]. The NAIA and NJCAA, governing bodies for collegiate athletics at other institutions, do not currently provide specific guidance to institutions about concussion education. However, even if concussion education is mandated, there can be variability in whether athletes do in fact receive any educational materials, and in the nature of these materials. Despite the NCAA’s mandate about concussion education, nearly one in four member institutions reported in 2013 that they did not provide their athletes with annual concussion education [42]. Even if institutions are in compliance, this does not mean that the educational effort is achieving its desired aims. A pilot study of the education provided to NCAA Division I men’s ice hockey players, all in compliance with the educational mandate, found substantial variability in what was delivered: some teams viewed a professionally produced video, listened to a lecture from the team’s Athletic Trainer and received a handout about concussion safety, while other teams received an email during the summer, ostensibly containing educational information, but that no team members recalled opening [41]. Perhaps not surprisingly, the authors found substantial variability in whether the concussion education that the athletes received was associated with any change in concussion knowledge or other cognitions predictive of concussion preventive behaviors. These findings underscore Finch and colleague’s recommendation that concussion guidelines place increased focus on how preventive programming is disseminated and implemented, rather than solely on the content [79].

Providennza and colleagues have described the importance of concussion knowledge translation strategies that meet the information needs and learning style preferences of target populations [80]. This means that a one-size-fits-all approach to concussion education is likely not appropriate, and underscores the importance for those designing and disseminating concussion education of being aware of the target population’s unique needs. Kroshus and colleagues recently surveyed collegiate athletes about their delivery preference for concussion education and found that athletes tended to prefer information delivered in lecture or video form [81]. While the team’s Athletic Trainer was overwhelmingly the preferred individual to be delivering this information, around half of respondents indicated that they would like their team coach to be involved in this process. Currently, coaches are rarely involved in delivering concussion education to athletes [81]. Critically, decisions about concussion education should be made by individuals with expertise relevant to concussion safety and not by coaches. However, engaging coaches in the delivery of this education (e.g., having them in the room when information is presented by the team’s athletic trainer, or having them provide their own editorial content endorsing the education provided by the team’s athletic trainer) may be an effective strategy to meet the learning preferences of athletes, while ensuring that the appropriate content is communicated. Engaging coaches in the education-delivery process could have the benefit of creating a perception among athletes that concussion safety is something valued by the team coach.

In addition to ensuring that the format of concussion education and the way it is delivered meets athlete preferences and learning needs, effective programming requires a systematic, typically theory-driven, approach to identifying and targeting cognitions that are predictive of the preventive behaviors that are being addressed. Finch has described how a reason that many sports injury prevention programs do not meet their behavioral goals is that they do not adequately incorporate an awareness of the target population’s unique context and they are not systematically designed to target the cognitions necessary to change behavior in that population [82]. Most existing concussion education programs for athletes that have published evaluations have focused on increasing knowledge about concussion symptoms, what should be done if an athlete suspects they have sustained a concussion and the importance of reporting symptoms of a concussion. A recent review by Caron and colleagues provides detail about the content and efficacy of these programs [83]. These programs have tended to be evaluated in terms of whether or not they change concussion knowledge, with relatively consistent short-term, but not lasting, changes in concussion knowledge. Behavioral changes have tended not to occur as a result of exposure to these programs or have not been measured in program evaluation.

A growing body of evidence about secondary prevention-focused concussion education for athletes is identifying cognitions that are predictive of reporting behavior. While the ability to recognize symptoms of a concussion is certainly necessary for reporting those symptoms, changing knowledge alone does not appear to be sufficient to reliably increase concussion reporting behavior [84]. The Theory of Planned Behavior (TPB) [85], an expectancy value theory in which an athlete’s attitudes about the expected consequences of performing a given behavior, what he or she thinks that important others would do and the control that the athlete believes he or she has overperformance of the behavior, has been proposed as a relevant theoretic framework for conceptualizing concussion education programming for athletes [86–88]. Prospective research indicates that perceived concussion reporting norms are a particularly important predictor of whether or not an athlete chooses to report their concussion, with individuals who have stronger athletic identity more likely to behave in ways that they believe ‘most athletes’ would behave [66]. Preliminary evidence suggests that, at least in some populations of athletes, norms may be misperceived, with athletes systematically tending to think that others have less safe attitude about concussion reporting than they themselves endorse. In other fields, such as alcohol education, a social norms approach, in which misperceived norms are corrected, has been a foundation for effective education programming. Concussion education informed by TPB, and addressing perceived social norms, should be developed and evaluated, and may represent the best potential, based on current knowledge, for positively impacting cognitions about the secondary prevention of harm from concussion among athletes. At a minimum, concussion education programming should identify behavioral targets, build on a well-explicated theory of behavior change and be evaluated and modified as necessary to ensure that it is in fact achieving its stated goal.

However, even if theory-driven concussion education for athletes is in fact developed and disseminated, there are limits to the potential effectiveness of these materials in the secondary prevention of harm from concussions. One reason is that education that aims to get athletes who have just sustained a concussion to recognize their symptoms and report their symptoms to a responsible adult is premised on the fact that the injured individual is able to make rational, deliberative decisions. But, in situations of high arousal or emotion, something that may frequently describe a game or practice situation [80–89], decision-making tends to be reactive and driven by emotional rather than deliberative risk calculations [90–92]. Consequently, while changing cognitions about the costs and benefits of reporting a concussion may change an athlete’s intention preinjury to report their symptoms, when in the heat of the moment the athlete’s calculus may be based on different inputs. Moreover, adolescent and young adult athletes do no not have a fully developed prefrontal cortex and have difficulty appropriately discounting future risks [93]. As such, risk-reduction approaches that are premised on athletes making rational calculations of short and long-term risks and benefits is a potentially flawed or at least an insufficient approach.

Another reason for the limits of athlete-focused concussion education is that behavior is constrained by the athlete’s context. Finch has described how effective sports injury prevention programming carefully considers the role that the athlete’s environment plays in facilitating or constraining the performance of preventive behaviors [82]. For example, if an athlete thinks their coach wants them to keep playing with symptoms of a suspected concussion, no amount of psychoeducation about the importance of reporting will fully offset this belief, should it in fact be true. A more successful approach would identify athlete cognitions that drive their preventive behaviors, and consider whether there are environmental changes that could help modify those cognitions. Recent evidence suggests that around one in four collegiate athletes experienced pressure during the previous season – from coaches, teammates, parents and fans – to continue playing while being symptomatic after a suspected concussion [76]. Currently, concussion education for NCAA coaches is recommended but not required; NAIA and NJCAA do not provide any guidance about coach education. The Centers for Disease Control and Prevention’s concussion education materials for coaches [94] have demonstrated effectiveness in youth and high school coaching populations [95,96]; research is needed to determine whether they are also effective in the college setting. It is critical to ensure that effective concussion education is developed for and targeted at athletes as well as other important stakeholders such as coaches. Coaches’ attitudes and behaviors play an important facilitating or constraining role on athlete preventive behaviors, and providing them with effective concussion education is critical for achieving the harm reducing promise of concussion education for athletes.

## Improvements

Existing evidence on concussion management practices and the effects of new concussion-related policies at non-NCAA college leagues is minimal. More research in this area is needed to ensure that athletes participating at these levels of competition are receiving adequate care in line with suggested best practices.

Although all college level sports leagues have in place policies related to concussion, the effectiveness of these policies is not well understood. Ostensibly, the goals of these policies include reducing the frequency and severity of acute concussive injury, improving rates of diagnosis where concussions do occur and optimizing the clinical care of athletes who are diagnosed with a concussion. Research examining whether and to what extent sports league concussion policies are achieving these goals is warranted and utilizing the empirical health law research framework [39] is suggested.

Concussion education should seek to do more than just raise awareness about concussions. Preventive behavioral targets should be specified, population-specific learning needs identified, the theory of behavior change explicated and programming iteratively evaluated and modified to ensure it is both efficacious in a controlled setting, and effective in a naturalistic setting.

The NCAA, NAIA and NJCAA should require that both athletes and coaches are adequately educated about concussion prevention. Mandates about education for these two stakeholder groups should be accompanied with educational programming that has demonstrated efficacy in changing the preventive behaviors identified by the respective associations as relevant targets.

Finally, much of the existing framework in the collegiate concussion management space is targeted toward secondary prevention: immediate removal from play, prompt diagnosis and appropriate management of the injury. As research relating to factors that can improve concussion injury prevention (primary prevention), these findings should be considered for incorporation in best practice guidelines and league-level policies.

## Conclusion & future perspective

Concussion management in college football has advanced in recent years, but there is still much room for improvement. Policies that have been put in place by sports leagues represent an important step in the right direction; however, empirically evaluating their effectiveness, and modifying appropriately to meet the intended goals of reducing the concussion burden in college athletes is critical. This includes both the existing secondary and tertiary prevention mechanisms as well as potential new primary prevention strategies. It is critical that approaches to prevention evolve as research on the health burden of brain trauma from contact sport grows. Although the concussive injury is a major focus in sports medicine and public health, the notion of a subconcussive injury has also been discussed [21,97] and implicated as causal in both acute [98,99] and long-term [29–36] health problems. Understanding the role of the cumulative forces, rather than just the concussive injuries, on acute and chronic health outcomes is critical for informing decisions about what constitutes the most impactful approaches for reducing the health risks of participation in contact and collision sports such as football. It is also important that approaches to risk reduction adapt should a substantial body of prospective and/or longitudinal data emerge about the association between acute concussive or subconcussive injury and later-life consequences of these injuries. In addition to the importance of making policy decisions that are informed by the evolving science on concussion, it is essential that education for athletes be also based on the best available evidence about the consequences of brain trauma from sport and the best available approaches for conveying such information. In sum, there are opportunities to reduce the variable implementation of concussion preventive approaches in collegiate football at the present time, and as research regarding the possible health consequences of concussion and strategies to reduce or eliminate this burden in athletes grows, education and policy should evolve to reflect this expanding body of knowledge.

**Executive summary**

- Given the large number of participants and comparatively high risk, examining concussion in American football is an important public health endeavor, though examination of other sports, including women’s sports, is also important.
- Within collegiate American football, rates of concussion vary across playing positions, time of year (spring vs fall football), and in what type of activity the athlete is participating (game vs practice).
- Given the acute and potential chronic health outcomes associated with concussion, minimizing the frequency and severity of these injuries is important.

**Policy**

- The National Collegiate Athletic Association (NCAA) put forth its Concussion Policy and Legislation in 2010 and suggested additional best practice guidelines in 2014. Rule changes affecting the way football is played at the college level have also been made.
- Existing evidence suggests broad though incomplete compliance with the existing NCAA concussion policy.
- Other sports leagues such as the National Association for Intercollegiate Athletics (NAIA) and National Junior College Athletics Association (NJCAA) have similar rules related to concussion as the NCAA, though their guidance is less robust.
- Evidence about the presence, implementation and efficacy of concussion management plans in non-NCAA college sports leagues is minimal. More research is needed.

**Clinical management**

- Clinical concussion management by NCAA clinicians has moved toward a multimodal approach for baseline testing, diagnosis/management and return-to-play decision-making.
- Recent research indicates that the frequency of computerized neuropsychological assessments has increased among NCAA clinicians in recent years.
- One study suggests that there has been a decrease in the use of symptom scales and comprehensive clinical evaluations, which, if true, should be rectified.
- Evidence about clinical management at non-NCAA colleges and junior colleges is sparse but suggests that management practices at at least some of these institutions may lag significantly behind best practice guidelines put forth by relevant clinician groups.
- The competitive environment of college sports may negatively influence concussion diagnosis and management, with multiple studies reporting the clinicians and athletes feel pressure to return to play prematurely.

**Education**

- Not all college sports leagues have mandates about concussion education for athletes, and even when a mandate is in place it is not always implemented, or may be implemented variably and with variable effectiveness. Although all states have concussion education mandates for athletes in high school and often younger, these mandates do not generally extend to college athletes.
- There are a range of primary, secondary and tertiary preventive behaviors that are potential targets for concussion education, and a range of stakeholders for whom concussion education could be targeted. Identifying behavioral outcomes, understanding population-specific learning needs, explicating a theory of behavior change on which program development will be premised and evaluating the programming are necessary steps to ensure concussion education achieves its implied goal.

**Areas of improvement**

- Although all college sports leagues concussion policies in place, the efficacy of these policies is not well understood. Most generally, college sports league guidance represents secondary and tertiary prevention efforts – that is, they aim to promptly evaluate, diagnose and appropriately manage injuries that occur. Additional efforts to reduce the frequency and severity of concussive injuries may be warranted. Future research empirically evaluating the effects of these policies is needed.
- Evidence regarding concussion management at non-NCAA college sports leagues is minimal and where it exists, suggests disparities in concussion management practices between NCAA schools and non-NCAA schools. Research in this area is warranted and where substantiated, improvement in disparities is of the utmost importance.
- To date, research examining the efficacy of concussion educational programs in changing concussion reporting behavior or cognitions predictive of concussion reporting behavior has produced mixed results. Using psychoeducational theory to build new and improved concussion education programs and employing appropriate empirical strategies to examine their efficacy in changing relevant behavioral outcomes is the next important step in this area.

**Conclusion & future perspective**

- Policies regarding concussion management in collegiate football have advanced in recent years, but there is still room to improve. Empirical evaluation of implementation and effectiveness of such policies is needed.
- Understanding the mechanisms between the acute concussive injury and possible later life health effects is critical as well as understanding the incidence and prevalence of these outcomes.
- As research increases, policy and education should follow accordingly.
